# Development of a cAMP biosensor-based assay for measuring serum bioactive concentrations of FSH

**DOI:** 10.1371/journal.pone.0342695

**Published:** 2026-02-13

**Authors:** Shiomi Ojima, Koichi Shinohara, Akihiko Wakatsuki, Kosuke Doi

**Affiliations:** 1 Research & Development Section, Diagnostic Division, Yamasa Corporation, Choshi, Chiba, Japan; 2 Department of Obstetrics and Gynecology, School of Medicine, Aichi Medical University, Nagakute, Aichi, Japan; University Hospital of Münster, GERMANY

## Abstract

Follicle-stimulating hormone (FSH) plays a pivotal role in reproductive physiology and is increasingly recognized for its involvement in the extragonadal system. The biological activity of FSH, which is modulated by glycosylation, varies with age, reproductive status, and pathological conditions. Although the serum FSH immunoreactive concentrations are measured routinely for patients with reproductive disorders, the significance of assessing the biological activity of FSH remains unclear. In this study, we aimed to develop a novel, rapid, and animal-free assay to quantify FSH bioactive concentrations using HEK293 cells co-expressing the human FSH receptor (FSHR) and a cyclic adenosine monophosphate biosensor. The assay demonstrated high specificity, reproducibility, and linearity, with minimal cross-reactivity with structurally related hormones. Application to clinical serum samples from postmenopausal women revealed a strong correlation between FSH immunoreactive and bioactive concentrations. Notably, estrogen replacement therapy resulted in a significant reduction in both FSH immunoreactive and bioactive concentrations, as well as in the FSH bioactive -to-immunoreactive concentration ratio, suggesting that FSH glycosylation patterns may have been altered, leading to a decrease in its bioactive concentrations. The findings collectively suggested that assessing FSH bioactive concentrations, in addition to immunoreactive concentrations, may provide further insights into hormonal regulation and its relevance to therapeutic evaluation. The biosensor-based assay could offer a practical and efficient tool for advancing our understanding of FSH function in both reproductive and non-reproductive contexts.

## Introduction

Follicle-stimulating hormone (FSH) is a gonadotropin secreted by the anterior pituitary gland that exerts various physiological effects through activation of the FSH receptor (FSHR), a member of the G protein-coupled receptor (GPCR) family. Upon activation, FSHR undergoes conformational changes that activate the heterotrimeric Gs protein, stimulate adenylyl cyclase, and subsequently increase intracellular cyclic adenosine monophosphate (cAMP) levels. This is a key signaling event through which FSH plays an essential role in reproductive function [1]. This cAMP-mediated signaling pathway underlies the physiological actions of FSH, including the promotion of follicular development and maturation in females [2] and the regulation of testicular development and spermatogenesis in males [3].

Serum FSH immunoreactive concentrations are routinely measured in clinical practice to diagnose reproductive disorders. In females, serum FSH is used to assess ovarian and hypothalamic amenorrhea, polycystic ovary syndrome (PCOS), and premature ovarian insufficiency (POI) [4]. Additionally, elevated FSH immunoreactive concentrations serve as a clinical biomarker for the diagnosis of menopause [5]. Serum FSH immunoreactive concentrations are used to assess testicular dysfunction in men [6]. In addition to its conventional role in the gonads, FSH regulates extragonadal physiological systems. Emerging evidence suggests that FSH is associated with bone metabolism [7], cardiovascular diseases [8], lipid metabolism [9], and neurodegenerative disorders [10]. Therefore, FSH has a broader biological significance than its traditional role in reproduction.

FSH is a 35-kDa glycoprotein composed of an α- and a β-subunit. The α-subunit, which is shared among glycoprotein hormones such as thyroid-stimulating hormone (TSH), luteinizing hormone (LH), and chorionic gonadotropin, contains two N-linked glycosylation sites. The β-subunit of FSH, which possesses a specific amino acid sequence and contains two N-linked glycosylation sites, determines its bioactivity. The structural characteristics of the α- and β-subunits, particularly the unique sequence and glycosylation of the β-subunit, determine the specificity and biological activity of FSH.

The physiological functions of FSH are modulated by the diversity of its glycosylation patterns. While the α-subunit of FSH is consistently glycosylated at both sites, glycosylation of the β-subunit is heterogeneous. The number of glycans on the β-subunit of FSH influences its circulatory half-life, receptor affinity, and downstream signal transduction. In addition to the number of glycans, structural diversity arises from differences in glycan branching and sialylation, which influence its binding affinity with the FSH receptor, circulatory half-life, and biological activity [11]. Since the structure of the β-subunit influences the biological activity of FSH, assessing its bioactive concentration is crucial for elucidating the functional implications of its molecular heterogeneity.

Assessing FSH bioactive concentrations could contribute remarkably to understanding its physiological functions and evaluating the impact of its glycosylation variants. Variations in FSH glycoforms have been reported in relation to the reproductive cycle [12], aging [13], and pathological conditions, such as PCOS [14], suggesting that FSH glycoforms may be involved in the pathophysiology of these conditions. Although glycoform profiling has traditionally relied on complex techniques, such as electrophoresis and mass spectrometry, bioactivity measurement offers a simpler alternative. Existing bioassays include animal-based methods, such as the Steelman–Pohley assay [15] and non-animal approaches, such as reporter gene assays [16]. However, both methods are time-consuming and technically demanding, limiting their clinical applicability. Therefore, a rapid and user-friendly assay system would be preferable to facilitate a broader evaluation of FSH bioactive concentrations.

A new approach could involve the use of a biosensor-based assay to enable the rapid and reliable assessment of FSH bioactive concentrations. A biosensor-based assay could utilize engineered cells co-expressing a GPCR and a cAMP biosensor, allowing the real-time detection of intracellular signaling events triggered by ligand binding. Such an assay has been successfully used to measure bioactive molecules, including thyroid-stimulating autoantibodies and arginine vasopressin [17,18]. Given that FSHR is a Gs coupled GPCR that increases the intracellular cAMP levels upon activation by FSH, we hypothesized that a similar biosensor-based strategy could be adapted to monitor FSH bioactive concentrations. Biosensor-based assays may overcome the limitations of the existing methods and offer a clinically applicable solution.

In this study, we aimed to establish a biosensor-based assay that would enable the monitoring of FSH bioactive concentrations using cells co-expressing human FSHR and a cAMP biosensor. We used this system to measure the bioactive concentrations of FSH in clinical specimens.

## Materials and methods

### Samples

The World Health Organization (WHO) international standard for human FSH (NIBSC code: 92/510) was purchased from Reference Material Institute for Clinical Chemistry Standards (Yokohama, Japan), along with human thyroid stimulating hormone (TSH; NIBSC code: 81/565), human chorionic gonadotropin (hCG; NIBSC code: 07/364), and human luteinizing hormone (LH; NIBSC code: 81/535). Prolactin was purchased from PeproTech Inc. (Cranbury, USA). Testosterone was purchased from Tokyo Chemical Industry Co., Ltd. (Chuo-ku, Japan). Follitropin-alfa (GONAL-f) was purchased from Merck Serono S.A. (Geneva, Switzerland). FSH-, LH-, and hCG-depleted human serum was purchased from Cone Bioproducts (Seguin, USA). D-Luciferin potassium salt and forskolin were purchased from FUJIFILM Wako Pure Chemical Corporation (Osaka, Japan).

### Specimens

Specimens were collected from 15 postmenopausal women before and after estrogen replacement therapy (n = 30) at the Department of Obstetrics and Gynecology at Aichi Medical University. This study was conducted using existing samples and information; therefore, individual consent from research participants was not obtained. Instead, information regarding the study was disclosed on our institution’s website, and potential participants were provided with an opportunity to opt out of inclusion in the research. In prior studies, participants had already written consent for the possible future use of their data and samples for research purposes. The study was approved by the Ethics Committee of Aichi Medical University on December 4, 2023 (approval number: 2023−168). Data collection for this retrospective study was conducted within the period from December 4, 2023, to March 31, 2024. The data were accessed for research purposes on March 28, 2024. The authors had not accessed any personally identifiable information of the participants during or after data collection.

A total of 15 postmenopausal women were given 0.625 mg/day of oral conjugated equine estrogen (CEE) (n = 5), 1.0 mg/day of oral 17β estradiol (E2) (n = 4), or 50 μg/day of transdermal 17βE2 (n = 6) for three months. The participants received either estrogen alone or estrogen with dydrogesterone at 5 mg/day.

### Cell culture

HEK293A cells (Thermo Fisher Scientific, USA) were cultured in high-glucose Dulbecco’s Modified Eagle Medium (DMEM) supplemented with GlutaMAX™ (GIBCO, USA) and 10% fetal bovine serum (GIBCO, USA) in a humidified incubator at 37 °C with 5% CO_2_.

### Establishment of stable cell lines

Plasmid transfection was performed using polyethyleneimine (PEIMAX; Polysciences, USA) as the transfection reagent. HEK293 cells were seeded in 6-well plates and transfected with 2 µg of linearized pGloSensor-22F. After 24 h, the cells were harvested and reseeded into 10 cm dishes, and 400 µg/mL Hygromycin B GOLD (InvivoGen, USA) was added after another 24 h. Drug-resistant colonies were isolated using cloning discs. Single colonies that exhibited increased luciferase activity upon stimulation with forskolin were selected as stable cAMP-biosensor cell lines.

Stable cell lines expressing the cAMP biosensor were screened based on the signal-to-background (S/B) ratio of relative light units (RLU). The S/B ratio was calculated by comparing the luminescence signals from forskolin-treated cells with those from vehicle-treated cells. Several clones exhibited high S/B ratios and the clone with the highest ratio was selected for subsequent experiments.

The stable cAMP biosensor cell lines were then seeded in 6-well plates and transfected with 2 µg of a linearized plasmid encoding human FSHR. After 24 h of transfection, the cells were reseeded into 10 cm dishes, and after another 24 h, 2 µg/mL Puromycin (Nacalai Tesque, Japan) and 400 µg/mL Hygromycin B GOLD were added. Drug-resistant colonies were isolated using cloning discs. Single colonies that exhibited increased luciferase activity upon stimulation with serially diluted GONAL-f were selected as stable cell lines that co-expressed FSHR and the cAMP biosensor.

Stable cell lines co-expressing FSHR and the cAMP biosensor were screened based on the S/B ratio of RLU. The S/B ratio was calculated by comparing the luminescence signals of rFSH-treated cells with those of vehicle-treated cells. Several clones exhibited high S/B ratios, and the one with the highest ratio was selected for subsequent experiments.

### GloSensor assay

The reaction buffer was prepared using Hanks’ balanced salt solution containing 1% bovine serum albumin (BSA) and 5 mM HEPES adjusted to pH 7.4. D-Luciferin potassium salt was dissolved to 100 mM in 10 mM HEPES buffer (pH 7.5) and subsequently added to the reaction buffer, hereafter referred to as the substrate-containing buffer. Standard solutions were prepared by adding GONAL-f to the FSH-, LH-, and hCG-depleted human serum. Standard solutions or test samples were diluted fourfold with the reaction buffer and mixed.

Stable cell lines co-expressing FSHR and GloSensor, stored at −80 °C (3.0 × 10^6^ cells/mL), were thawed in a 25 °C water bath for 10 min with gentle shaking. The thawed cells were added to the substrate-containing buffer and mixed to prepare a cell suspension (1.5 × 10^5^ cells/mL). Fourfold diluted human serum samples (20 µL) were added to a 96-well white plate, followed by 100 µL of the cell suspension, and incubated at 25 °C for 30 min. RLU were measured using a GloMax Explorer luminometer (Promega, USA).

### Statistical analysis

Correlation plots were generated using GraphPad Prism 10 (GraphPad, UK), and Pearson’s correlation coefficients were calculated to assess the relationship between serum FSH immunoreactive concentrations and FSH bioactive concentrations.

Comparisons between samples collected before and after estrogen replacement therapy were performed using the paired Student’s t-test. Differences were considered statistically significant at *P* ≤ 0.05.

## Results

### Establishment of a stable cell line expressing FSHR and a cAMP biosensor for measuring FSH bioactive concentrations

**Fig 1:**
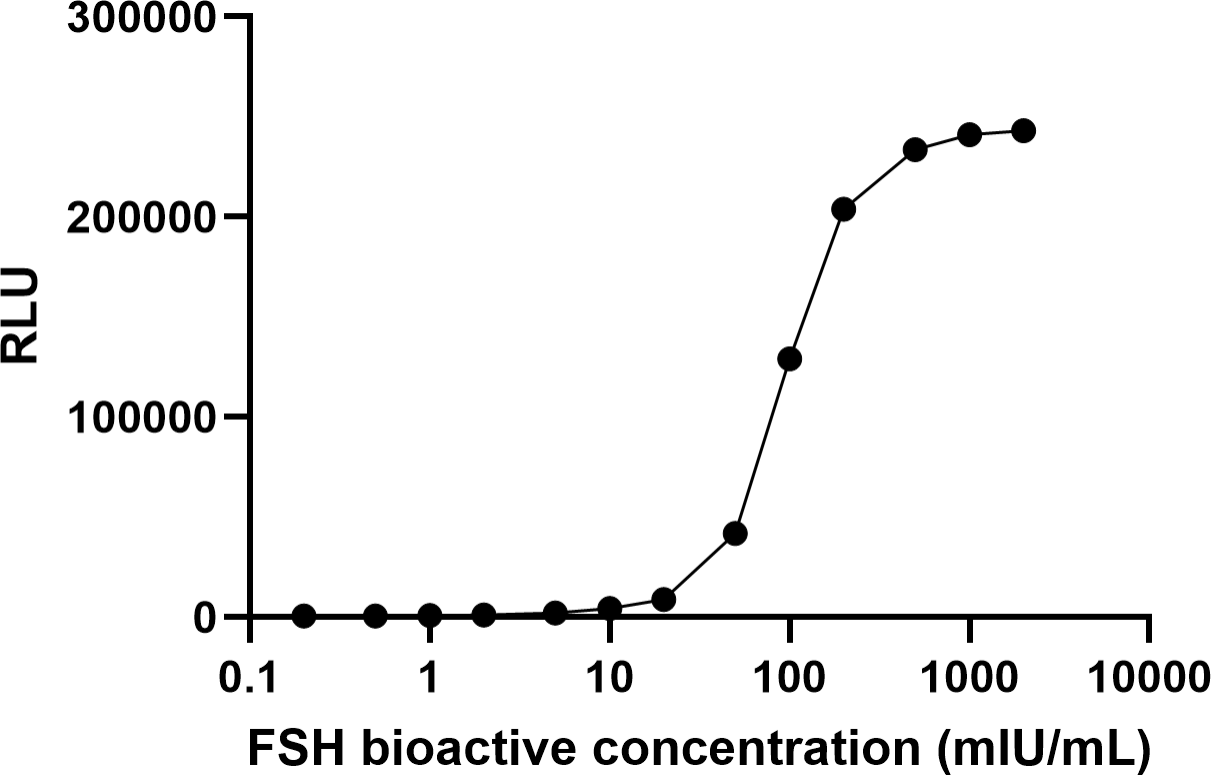
Measurement of recombinant FSH using stable cell lines co-expressing FSHR and cAMP biosensor. Recombinant FSH (GONAL-f) was quantified using stable cell lines co-expressing the human FSH receptor (FSHR) and GloSensor cAMP biosensor. FSH stimulation induced a concentration-dependent increase in luminescence, indicating activation of the cAMP signaling pathway.

To establish a HEK293 cell line co-expressing the human FSHR (hFSHR) and a cAMP biosensor, we transfected two plasmids encoding hFSHR and the GloSensor-22F cAMP biosensor into HEK293 cells. Stable clones were selected from the hygromycin B- and puromycin-resistant pools using cloning disks. The resulting cell lines were treated with serial dilutions of rFSH (GONAL-f, Follitropin-alfa) to evaluate their responsiveness. The selected clone exhibited a robust luciferase response following FSH stimulation (Fig 1), indicating its suitability for application in a biosensor-based assay system for detecting FSH activity.

### Assay system validation

The reliability and robustness of the newly developed biosensor-based assay were evaluated through validation studies that focused on reproducibility, linearity, and potential interference from coexisting substances.

Intra-assay reproducibility was assessed using 10 replicate measurements for each of the three human serum specimens. The mean FSH bioactive concentration values for samples 1, 2, and 3 were 75.3, 35.7, and 8.5 mIU/mL, respectively, with coefficients of variation (CV%) of 4.8%, 5.5%, and 4.2%, respectively (Table 1).

**Table 1:** Intra-assay and inter-assay variations of the FSH bioassay.

Intra-assay	Sample 1	Sample 2	Sample3
N	10	10	10
Mean (mIU/mL)	75.3	35.7	8.5
SD (mIU/mL)	3.6	2.0	0.4
CV (%)	4.8	5.5	4.2
Inter-assay	Sample 1	Sample 2	Sample3
N	3	3	3
Mean (mIU/mL)	72.4	35.2	8.7
SD (mIU/mL)	3.8	1.6	0.3
CV (%)	5.3	4.6	3.7

Intra- and inter-assay variations were assessed by the same operator on the same day.

Inter-assay reproducibility was evaluated across three independent experiments for each specimen, yielding mean FSH bioactive concentration values of 72.4, 35.2, and 8.7 mIU/mL, with CV% of 5.3%, 4.6%, and 3.7%, respectively.

**Fig 2:**
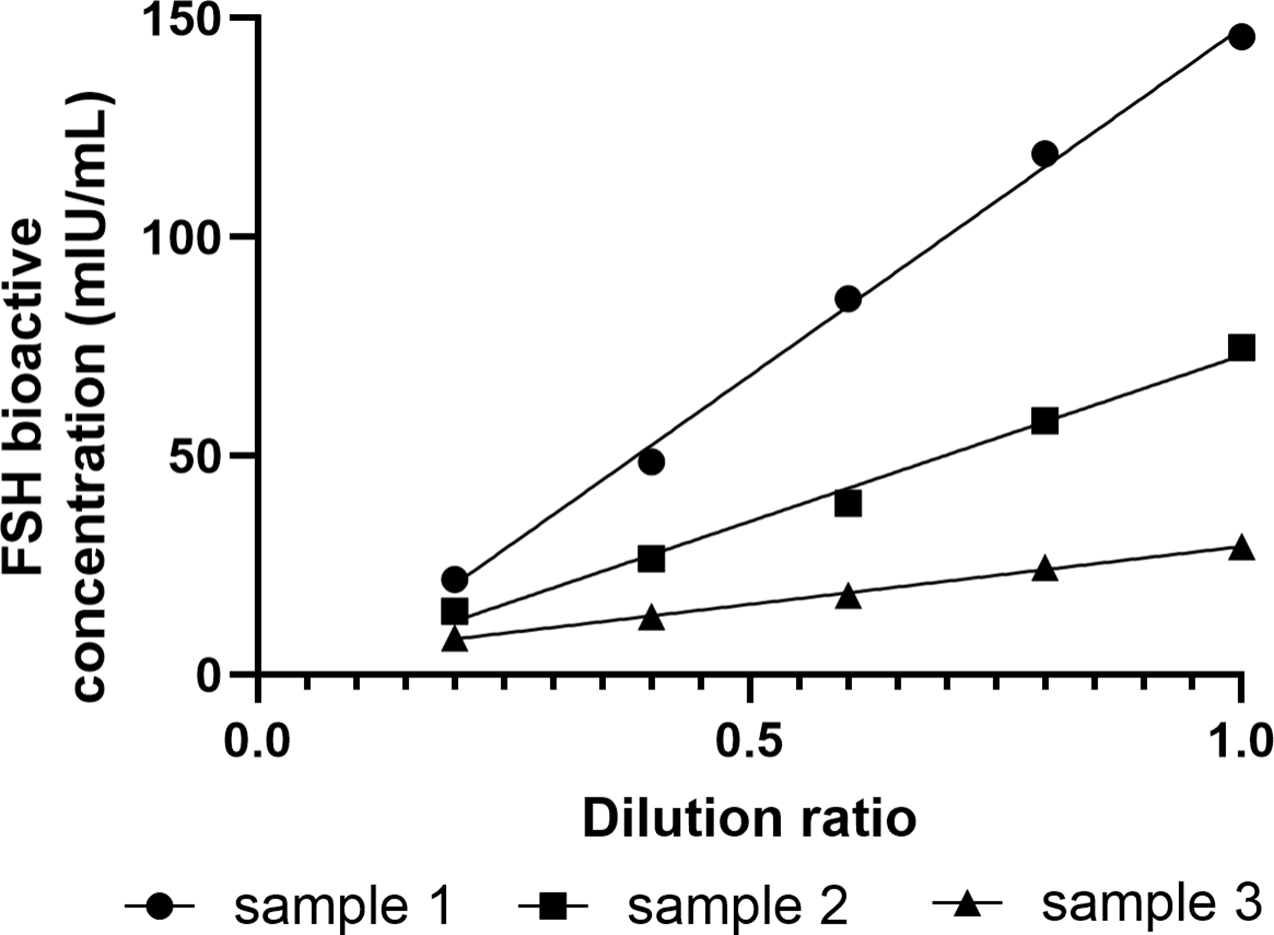
Dilution linearity in the FSH bioassay. The samples were serially diluted with FSH-depleted human serum to assess the dilution linearity of the assay. All three specimens demonstrated good linearity in FSH bioactive concentrations.

Linearity of the assay was evaluated by measuring human serum samples that were serially diluted in FSH-depleted serum. All three samples demonstrated good linearity in FSH bioactive concentrations across the dilution range (Fig 2).

Specificity of the assay was evaluated by testing five hormones, namely TSH, LH, hCG, prolactin, and testosterone. TSH, LH, and hCG were selected due to their structural similarity with FSH, since they are glycoprotein hormones sharing a common α-subunit. Prolactin and testosterone were included, since they are involved in reproductive function and may be present at elevated levels in certain clinical conditions. Each hormone was tested at supraphysiological concentrations to evaluate any potential interference. When tested with TSH (50 µIU/mL) and LH (500 mIU/mL), the measured FSH bioactive concentrations was 2.3 mIU/mL and 0.9 mIU/mL, respectively. No detectable signal was observed for hCG (25,000 mIU/mL), prolactin (500 ng/mL), or testosterone (1 ng/mL) (Table 2). The results indicated that the assay exhibited minimal cross-reactivity with other hormones, supporting its specificity for measuring FSH bioactive concentrations.

**Table 2:** Effects of coexisting substances on FSH bioactive concentrations measurement.

	Concentration of tested hormone	FSH bioactive concentrations
TSH	50 uIU/mL	2.3 mIU/mL
LH	500 mIU/mL	0.9 mIU/mL
hCG	25000 mIU/mL	N.D.
Prolactin	500 ng/mL	N.D.
Testosterone	1 ng/mL	N.D.

The potential interference of coexisting substances was evaluated using TSH (50 µIU/mL), LH (500 mIU/mL), hCG (25,000 mIU/mL), prolactin (500 ng/mL), and testosterone (1 ng/mL). Measured values for TSH and LH were 2.3 mIU/mL and 0.9 mIU/mL, respectively. The hCG, prolactin, and testosterone levels were below detection limits. N.D., not detected.

The findings demonstrated that the developed assay system could provide specific and reproducible quantification of FSH bioactive concentrations in serum, with minimal cross-reactivity with other hormones.

### Measurement of clinical specimens

**Fig 3:**
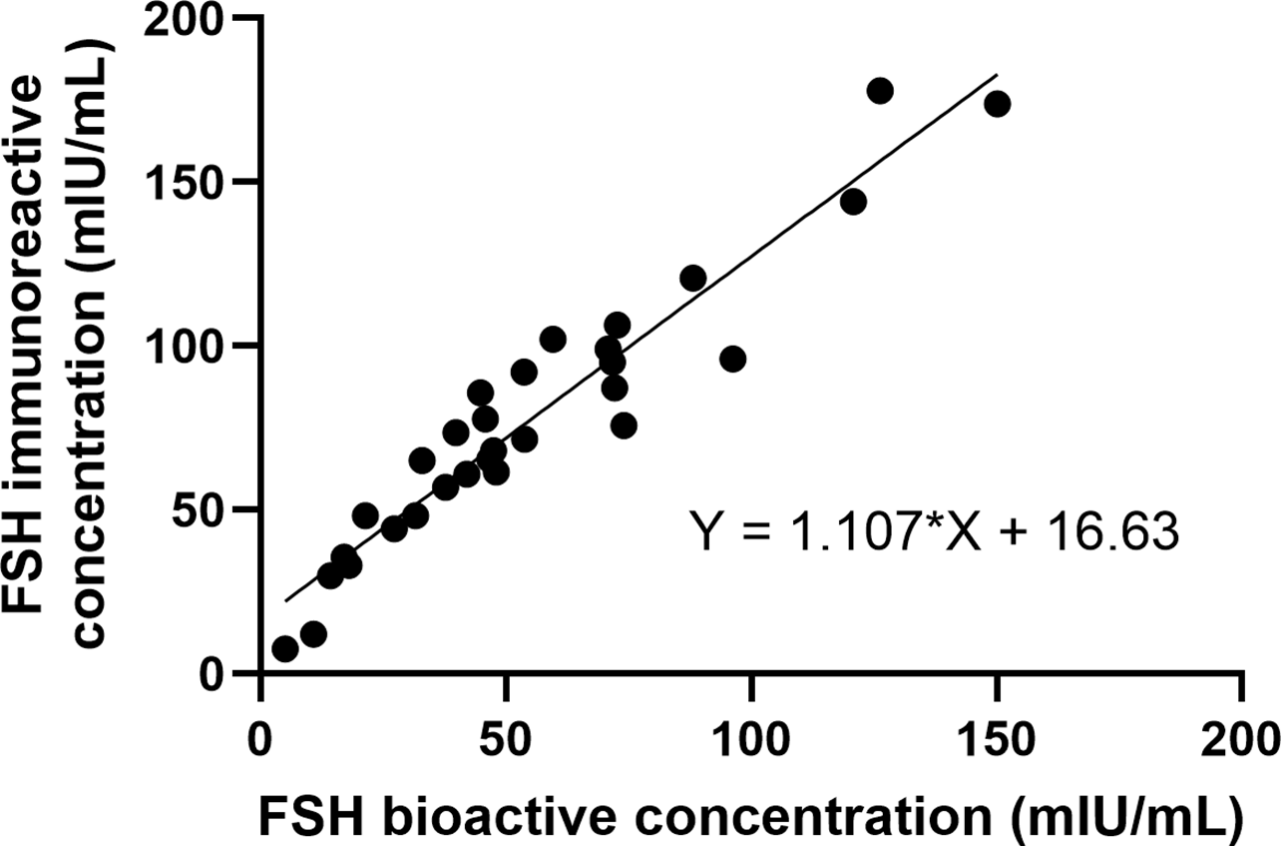
Correlation between serum FSH immunoreactive concentrations and FSH bioactive concentrations. The relationship between serum FSH immunoreactive concentrations and FSH bioactive concentrations was evaluated in 30 postmenopausal women. A strong positive correlation was observed with a slope of 1.107 and a correlation coefficient of r = 0.955.

We analyzed 30 serum specimens from postmenopausal women to investigate the relationship between the immunoreactive concentrations and bioactive concentrations of FSH (Fig 3). A strong correlation was observed between the two, with a slope of 1.107 and a correlation coefficient of 0.955.

Among the 30 samples analyzed, 15 were obtained prior to the initiation of estrogen replacement therapy, whereas the other 15 were collected following the commencement of the therapy. Further analysis of the two groups revealed that the mean FSH bioactive concentrations (expressed as mean ± standard deviation) was 74.1 ± 35.9 mIU/mL before therapy and 35.2 ± 21.3 mIU/mL after therapy, the latter showing a significant decrease (*p* = 0.002) (Table 3). The mean FSH immunoreactive concentrations in each group was 99.1 ± 39.7 mIU/mL and 55.3 ± 28.7 mIU/mL, respectively, showing a significant decrease after therapy (*p* = 0.003). On the other hand, the mean E2 in each group was 28.3 ± 47.7 pg/mL and 62.1 ± 58.0 pg/mL, respectively, indicating an upward trend that did not reach statistical significance (*p* = 0.132). The mentioned results were largely consistent with previous reports of reduced FSH immunoreactive concentrations and enhanced E2 concentrations during estrogen replacement therapy in postmenopausal women [19]. Furthermore, given the strong correlation between FSH bioactive concentrations and immunoreactive concentrations, the observed decrease in FSH bioactive concentrations was likely attributable to a reduction in serum FSH immunoreactive concentrations. E2 levels typically increase during estrogen replacement therapy. In our analysis, two samples (No. 4 and 15) exhibited elevated E2 concentrations before the initiation of hormone therapy (S1 Table). This suggested that the individuals were likely in the perimenopausal stage rather than postmenopausal. Therefore, an absence of a significant difference in the serum E2 levels before and after hormone treatment in the patients could be attributable to their transitional hormonal status.

**Table 3:** Comparison of age, serum FSH immunoreactive concentrations, FSH bioactive concentrations, FSH ratio, and E2 levels before and after E2 replacement therapy.

	Pre	Post	p-value
Age (years, Mean±SD)	48.4 ± 4.3		
FSH bioactive concentrations (mIU/mL, Mean±SD)	74.1 ± 35.9	35.2 ± 21.3	0.002
FSH immunoreactive concentrations (mIU/mL, Mean±SD)	99.1 ± 39.7	55.3 ± 28.7	0.003
FSH ratio (Mean±SD)	0.73 ± 0.14	0.63 ± 0.13	0.048
E2 (pg/mL, Mean±SD)	28.3 ± 47.7	62.1 ± 58.0	0.132

In postmenopausal women, E2 replacement therapy significantly reduced both serum FSH immunoreactive and bioactive concentrations, while serum E2 levels showed an upward trend that did not reach statistical significance (*p* = 0.132). In addition, FSH bioactive concentrations, FSH immunoreactive concentrations, and the FSH ratio (defined as FSH bioactive concentrations per unit FSH immunoreactive concentrations) significantly decreased following the therapy.

**Fig 4:**
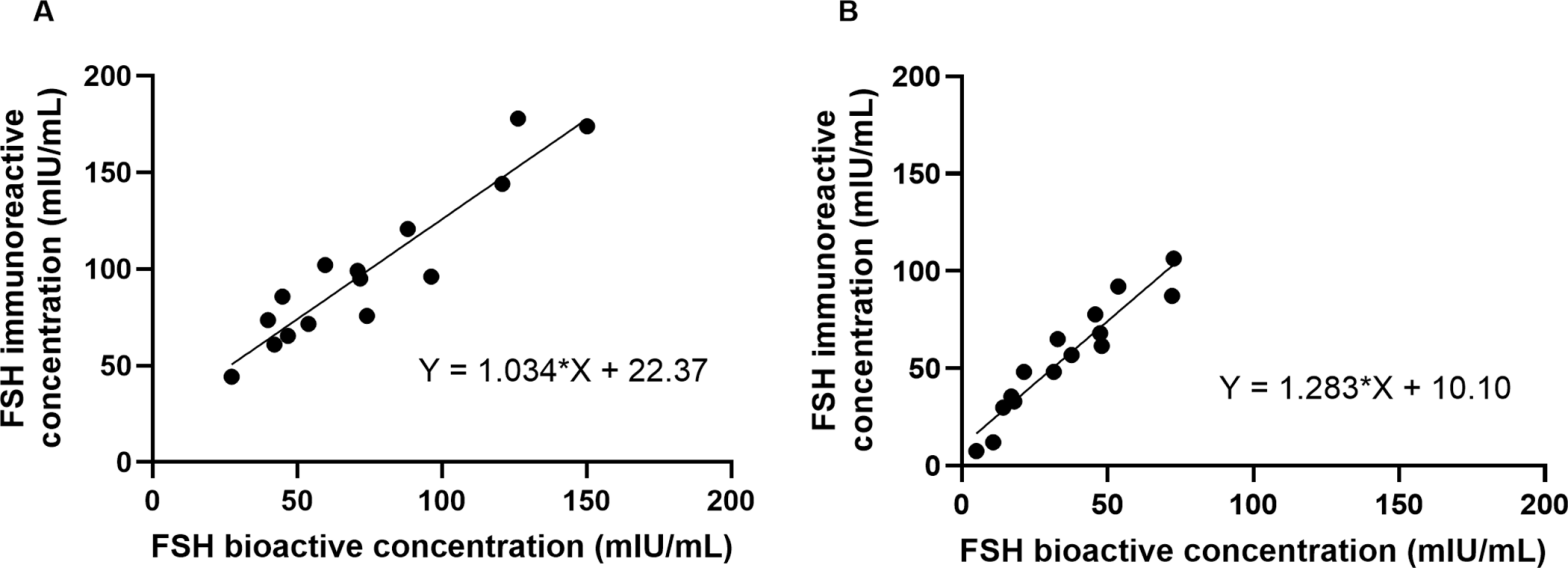
Correlation between serum FSH immunoreactive concentrations and FSH bioactive concentrations before and after E2 replacement therapy in postmenopausal women. In postmenopausal women, the correlation between serum FSH immunoreactive concentrations and bioactive concentrations was analyzed before **(A)** and after **(B)** E2 replacement therapy. The slope of the regression line was 1.034 before and 1.283 after therapy, indicating a shift in their relationship after treatment.

The mean FSH ratio (defined as FSH bioactive concentrations per unit of FSH immunoreactive concentrations) was 0.73 ± 0.14 before therapy and 0.63 ± 0.13 after therapy, indicating a significant decrease (*p* = 0.048) (Table 3). The correlation between FSH bioactive concentrations and FSH immunoreactive concentrations before estrogen replacement therapy had a slope of 1.034 and a correlation coefficient of r = 0.935 (Fig 4A). After estrogen replacement therapy, the correlation had a slope of 1.283 and a correlation coefficient of r = 0.950 (Fig 4B). The results collectively suggested that estrogen replacement therapy not only decreases FSH immunoreactive concentrations but also the FSH ratio.

The findings suggested that, although FSH immunoreactive concentrations and bioactive concentrations are highly correlated, FSH-specific activity may vary depending on the physiological or treatment status of the sample group.

## Discussion

To evaluate the specificity of the assay, interference testing was conducted using supraphysiological concentrations of several hormones, including TSH, LH, prolactin, testosterone, and hCG. Among them, only TSH and LH caused measurable increases in apparent FSH activity, whereas prolactin, testosterone, and hCG did not produce any detectable signal. The cross-reactivity is likely due to the structural similarity among the glycoprotein hormones, since FSH, LH, and TSH share a common α-subunit, which may allow partial binding to the FSHR under high concentrations. TSH levels in healthy females typically fall within the range of 0.45–4.12 µIU/mL [20], but can be significantly elevated in cases of hypothyroidism. LH levels typically range from 9.1–62.2 mIU/mL during the ovulatory peak and 10.3–50.0 mIU/mL in postmenopausal women [21], but might exceed these ranges in individuals with PCOS. In disorders characterized by elevated hormone levels, FSH bioactive concentrations may be overestimated, requiring cautious interpretation of the results. Nevertheless, since the tested concentrations of TSH (50 µIU/mL) and LH (500 mIU/mL) far exceeded normal physiological levels, the assay is expected to perform reliably in most clinical specimens. We intend to optimize the current assay system to minimize or eliminate cross-reactivity with TSH and LH in future studies, potentially through receptor modification or the addition of antibodies targeting interfering hormones [17,18].

We hypothesized that measuring FSH bioactive concentrations could reveal a novel clinical utility of FSH beyond its conventional role. The glycoforms of FSH vary depending on the reproductive cycle, age, and pathological conditions, such as PCOS [12–14]. If FSH alters its glycoform in response to diverse physiological conditions, such variations could occur in diseases other than PCOS as well. Although FSH glycoform profiling is performed using electrophoresis or mass spectrometry [13], we consider FSH assays for FSH bioactive concentrations to be more practical and accessible for evaluating the changes. If FSH bioactive concentrations can be assessed easily, evaluation of the disease would be possible in a manner that reflects the heterogeneity of FSH.

Our newly developed assay could provide a simple and time-efficient alternative to existing methods for evaluating FSH bioactive concentrations. The classical Steelman–Pohley assay, which measures FSH-induced ovarian weight gain in rats, is considered the gold standard; however, it requires approximately three days, involves high inter-animal variability, and raises ethical concerns due to animal use. As an animal-free alternative, a reporter gene assay system was developed to evaluate rFSH [16]. Although the system enabled accurate quantification of FSH bioactive concentrations, it required 16–24 h of cell pre-culture followed by an additional 4 h of sample incubation for the assay procedure. Extended hands-on time presents a challenge for its implementation in clinical settings. In contrast, our newly developed biosensor-based assay could complete the entire measurement process within approximately 1 h, from cell thawing to signal detection. The rapid and convenient workflow would offer significant advantages over previously reported methods. The Steelman–Pohley assay allows the evaluation of FSH bioactive concentrations considering its circulatory half-life. In contrast, the biosensor-based assay is inherently unsuitable for such an evaluation since it was developed specifically for measuring FSH bioactive concentrations in human serum. Therefore, selecting the appropriate method based on the intended purpose would be important.

Serum levels of FSH have been reported to influence lipid and bone metabolism and cognitive function in Alzheimer’s disease. Blocking FSH reduces serum cholesterol levels and increases bone mass [9,22]. In addition, anti-FSH antibodies have been reported to prevent the onset of Alzheimer’s disease [10].

In this study, we developed a novel technique for measuring FSH bioactive concentrations and showed that hormone therapy, such as estrogen alone or estrogen with progestin, in postmenopausal women decreased both FSH bioactive concentrations and FSH immunoreactive concentrations that were elevated after menopause. The half-life of pituitary gonadotropins, such as LH and FSH, is increased by sialylation in women [23]. Administration of estradiol benzoate after oophorectomy significantly reduced the mRNA levels of 2,3-sialyltransferase, an enzyme that incorporates sialic acid residues into FSH [24]. This suggests that estrogen may shorten the half-life of FSH. In the present study, we demonstrated that hormone therapy reduced not only serum FSH immunoreactive concentrations but also FSH bioactive concentrations, indicating that estrogen might reduce the sialylation of FSH and consequently shorten its half-life. Hormone therapy improves lipid and bone metabolism. Additionally, a study by Nerattini et al. showed that hormone therapy initiated during midlife reduced the risk of dementia, whereas late-life use did not [25]. Therefore, estrogen-induced reductions in FSH bioactive concentrations and serum FSH immunoreactive concentrations could affect lipid and bone metabolism and the risk of Alzheimer’s disease. The findings highlighted the importance of evaluating not only the immunoreactive concentrations, but also the bioactive concentrations of FSH. Since FSH bioactive concentrations is modulated by its glycosylation status, which can vary with physiological and pathological conditions, measuring bioactive concentrations might provide insights that immunoreactive levels alone cannot.

The glycosylation patterns of FSH are influenced by factors such as aging, the reproductive cycle, and disorders such as PCOS. In our study, hormone therapy led to a reduction in the FSH ratio (bioactive concentrations per unit FSH immunoreactive concentrations), suggesting a shift in glycosylation and biological potency. Despite this novel observation, our analysis was limited to postmenopausal women receiving hormone replacement therapy. Previous studies had reported glycosylation-related changes in FSH levels in patients with PCOS; similar alterations could occur in other reproductive disorders, such as POI, amenorrhea, and infertility. Although we were unable to include these conditions in the present study due to limited sample availability, we plan to explore the clinical relevance of FSH bioactive concentrations in these contexts in future. Future studies assessing the bioactive concentrations of FSH might extend its clinical relevance beyond reproductive disorders. Given the emerging evidence of the involvement of FSH in conditions such as osteoporosis, atherosclerosis, hyperlipidemia, and Alzheimer’s disease, such evaluations could provide valuable insights into both reproductive and non-reproductive pathologies, thereby enhancing our understanding and management of a wide range of diseases.

In summary, the newly developed biosensor-based assay could provide a rapid and straightforward method for measuring FSH bioactive concentrations. The assay system enabled the analysis of clinical specimens within 1 h, offering a significantly more convenient approach than conventional assays for determining FSH bioactive concentrations. Evaluation of postmenopausal serum samples revealed a decrease in the FSH ratio following hormone replacement therapy. The finding could shed light on the previously unrecognized aspects of FSH that could not otherwise be assessed using conventional immunoreactive concentration-based measurements. In future studies, we plan to analyze specimens from various disorders, including PCOS, that have been reported to exhibit altered FSH glycosylation patterns, to further investigate the clinical utility of measuring FSH bioactive concentrations.

## Supporting information

S1 Table FSH bioactive concentrations, immunoreactive concentrations, ratio, and E2 levels in individual serum samples before and after therapy. (DOCX)

S1 File Source data of this study. (XLSX)
