# Supplementary material for: Development of a cAMP biosensor-based assay for measuring serum bioactive concentrations of FSH
Source: PLoS One. 2026 Feb 13;21(2):e0342695. doi: 10.1371/journal.pone.0342695 (PMC12904449; doi:10.1371/journal.pone.0342695)
Supplement: S1 Table — (DOCX) [file pone.0342695.s001.docx]

**Supporting information**

**S1 Table. FSH bioactive concentrations, immunoreactive concentrations, ratio, and E2 levels in individual serum samples before and after therapy.**

|  | FSH  bioactive concentration | | FSH immunoreactive concentration | | FSH ratio | | E2 | |
| --- | --- | --- | --- | --- | --- | --- | --- | --- |
|  | (mIU/mL) | | (mIU/mL) | |  |  | (pg/mL) | |
|  | Pre | Post | Pre | Post | Pre | Post | Pre | Post |
| No.1 | 70.8 | 10.8 | 99.0 | 12.1 | 0.71 | 0.89 | 11 | 247 |
| No.2 | 44.8 | 5.0 | 85.8 | 7.6 | 0.52 | 0.66 | 22 | 36 |
| No.3 | 120.8 | 45.8 | 144.0 | 77.8 | 0.84 | 0.59 | 10 | 133 |
| No.4 | 39.8 | 72.7 | 73.6 | 106.4 | 0.54 | 0.68 | 104 | 11 |
| No.5 | 126.3 | 21.4 | 177.9 | 48.2 | 0.71 | 0.44 | 10 | 35 |
| No.6 | 88.2 | 14.3 | 120.7 | 29.9 | 0.73 | 0.48 | 10 | 24 |
| No.7 | 27.3 | 37.7 | 44.2 | 56.9 | 0.62 | 0.66 | 10 | 51 |
| No.8 | 53.8 | 18.0 | 71.6 | 33.1 | 0.75 | 0.54 | 10 | 39 |
| No.9 | 74.0 | 48.1 | 75.8 | 61.5 | 0.98 | 0.78 | 10 | 50 |
| No.10 | 96.3 | 47.5 | 96.1 | 68.1 | 1.00 | 0.70 | 10 | 51 |
| No.11 | 150.2 | 53.7 | 173.9 | 92.0 | 0.86 | 0.58 | 11 | 63 |
| No.12 | 71.7 | 31.6 | 95.1 | 48.2 | 0.75 | 0.66 | 10 | 50 |
| No.13 | 46.7 | 17.0 | 65.4 | 35.5 | 0.71 | 0.48 | 10 | 64 |
| No.14 | 59.6 | 32.9 | 102.0 | 65.1 | 0.58 | 0.51 | 10 | 29 |
| No.15 | 42.0 | 72.2 | 60.9 | 87.2 | 0.69 | 0.83 | 177 | 48 |

Individual values of serum FSH bioactive concentrations, FSH immunoreactive concentration, FSH ratio (defined as FSH bioactive concentrations per unit FSH immunoreactive concentrations), and estradiol (E2) levels in 15 postmenopausal women before and after estrogen replacement therapy.
